# Glances at the Hospitals

**Published:** 1905-12-09

**Authors:** 


### GLANCES AT THE HOSPITALS.

### ROYAL HANTS HOSPITAL AT WINCHESTER.

The total ordinary income amounted to £5,267, and the total ordinary expenditure to £6,606, showing a deficit of £1,339 on the year's working. This is partly accounted for by a falling off in the amount of the annual subscriptions, but is chiefly owing to the increase in the number of in-patients and to the higher prices of provisions. The daily average of beds occupied was eighty-three, the average cost per bed occupied was £67 10s., the average cost of each in-patient was £5 10s., and the average cost per patient was 4s. 1d. a day. The average stay of each patient in the hospital was twenty-seven days. At the beginning of the year 76 patients were resident in the wards, and 966 were admitted during it, making a total of 1,042 under treatment. Of these 527 were discharged recovered, 199 much improved, 106 improved, 63 for other reasons, 47 died, and 100 remained in the wards. The medical and surgical tables are quite useless, and might as well have been left out, as they merely show the number of patients treated for the diseases named, and such figures without results of treatment can serve no useful purpose. There is no anæsthetic table.

### CARDIFF INFIRMARY.

The income was £12,176, and the expenditure was £11,805, so that there was a balance of £370 in favour of the year's working; but additions and improvements are in progress, and the current account was drawn on to meet some of the expenses thereof. We are glad to note that one-third of the voluntary income was subscribed by the workpeople. The average daily number resident was 145, and average daily cost of each in-patient was 3s. 7d. The number of beds available during the year was 154, the number of in-patients was 1,919, and were classified as 1,004 surgical, 272 medical, 370 ophthalmic, and 273 gynæcological. Of the 1,919 there were discharged cured 747, relieved 736, discharged for various reasons 120, died 164, and remaining in the institution at the end of the year 152. The average of each patient's stay in the wards was nearly twenty-eight days, the average cost per bed occupied was £65 5s., and the average daily cost per head for provisions was nearly 10d. Many useful additions have lately been made to this infirmary. The fire appliances have been added to; electric light has been installed, and the building has been placed in telephonic communication with the fire station. The scheme of heating has been completed, new accident wards have been opened, and also a new pavilion which contains a Finsen lamp-room and a Röntgen-ray apparatus. The medical and surgical tables are divided into four columns, showing the numbers "cured," "died," "remaining," and the "totals." The operation table shows a total of 528 operations, with thirty-six deaths. There were twenty-three amputations, with one death; the operation for appendicitis was performed twenty-six times, with five deaths; nephro-lithotomy four times, all being successful. A like result was obtained in four cases of suprapubic cystotomy. The anæsthetic table tells us that there were 1,373 administrations, chloroform being the agent in 574 cases, ether in 137, chloroform and ether mixed in 360, gas, ether, and chloroform in sequence in 88, A.C.E. in 14, kelene in 66, and nitrous oxide in 134.

